# Implementing the package of CDC and WHO recommended linkage services: Methods, outcomes, and costs of the Bukoba Tanzania Combination Prevention Evaluation peer-delivered, linkage case management program, 2014-2017

**DOI:** 10.1371/journal.pone.0208919

**Published:** 2018-12-13

**Authors:** Duncan MacKellar, Haruka Maruyama, Oscar Ernest Rwabiyago, Claire Steiner, Haddi Cham, Omari Msumi, Rachel Weber, Gerald Kundi, Chutima Suraratdecha, Tewodaj Mengistu, Johnita Byrd, Sherri Pals, Eliufoo Churi, Caitlin Madevu-Matson, Kokuhumbya Kazaura, Fernando Morales, Thomas Rutachunzibwa, Jessica Justman, Anath Rwebembera

**Affiliations:** 1 Division of Global HIV and TB, National Center for Global Health, Centers for Disease Control and Prevention, Atlanta, Georgia, United States of America; 2 ICAP at Columbia University, Dar es Salaam, Tanzania; 3 Centers for Disease Control and Prevention, Dar es Salaam, Tanzania; 4 CTS Global, Inc., assigned to Centers for Disease Control and Prevention, Dar es Salaam, Tanzania; 5 ICF International, Atlanta, Georgia; 6 ICAP at Columbia University, New York, New York, United States of America; 7 MoHCDGEC, Bukoba, Tanzania; 8 National AIDS Control Program, MoHCDGEC, Dar es Salaam, Tanzania; Boston University School of Public Health, UNITED STATES

## Abstract

Although several studies have evaluated one or more linkage services to improve early enrollment in HIV care in Tanzania, none have evaluated the package of linkage services recommended by the Centers for Disease Control and Prevention (CDC) and the World Health Organization (WHO). We describe the uptake of each component of the CDC/WHO recommended package of linkage services, and early enrollment in HIV care and antiretroviral therapy (ART) initiation among persons with HIV who participated in a peer-delivered, linkage case management (LCM) program implemented in Bukoba, Tanzania, October 2014 –May 2017. Of 4206 participants (88% newly HIV diagnosed), most received recommended services including counseling on the importance of early enrollment in care and ART (100%); escort by foot or car to an HIV care and treatment clinic (CTC) (83%); treatment navigation at a CTC (94%); telephone support and appointment reminders (77% among clients with cellphones); and counseling on HIV-status disclosure and partner/family testing (77%), and on barriers to care (69%). During three periods with different ART-eligibility thresholds [CD4<350 (Oct 2014 –Dec 2015, n = 2233), CD4≤500 (Jan 2016 –Sept 2016, n = 1221), and Test & Start (Oct 2016 –May 2017, n = 752)], 90%, 96%, and 97% of clients enrolled in HIV care, and 47%, 67%, and 86% of clients initiated ART, respectively, within three months of diagnosis. Of 463 LCM clients who participated in the last three months of the rollout of Test & Start, 91% initiated ART. Estimated per-client cost was $44 United States dollars (USD) for delivering LCM services in communities and facilities overall, and $18 USD for a facility-only model with task shifting. Well accepted by persons with HIV, peer-delivered LCM services recommended by CDC and WHO can achieve near universal early ART initiation in the Test & Start era at modest cost and should be considered for implementation in facilities and communities experiencing <90% early enrollment in ART care.

## Introduction

The considerable benefits of ART to reduce HIV-related morbidity and mortality, and HIV transmission risk to uninfected sexual partners and offspring are now well established [1–3]. To achieve HIV epidemic control, the United Nations AIDS Program established goals by the end of 2020 for countries to diagnose ≥90% of HIV-infected persons, initiate and retain ≥90% of diagnosed persons on ART, and achieve viral load suppression on ≥90% of persons on ART (90-90-90) [4]. Achieving optimal impact of the second and third “90” on reducing HIV transmission risk and HIV morbidity and mortality, however, hinges on early ART initiation following diagnosis [1,2]. In sub-Saharan Africa, although HIV testing and care and treatment programs have increased access to ART remarkably, many HIV-infected persons still initiate ART late in the course of their HIV disease [3,5,6]. Delayed enrollment in HIV care following diagnosis is a particularly important factor contributing to late ART initiation, particularly among young adults, men, and persons diagnosed in community settings [1,3,6–9].

To improve early enrollment in care and ART initiation following HIV diagnosis, CDC and WHO released recommendations on linkage services in 2014 and 2016, respectively [1,10]. No studies in Tanzania, however, have evaluated whether providing the CDC/WHO package of recommended services can routinely achieve ≥90% early enrollment in HIV care overall, and for groups that are more likely to delay enrollment in care. Evaluating the effects and costs associated with implementing the package of recommended linkage services is fundamental for informing scale up of these services. This evaluation is particularly important in light of recent WHO recommendations to initiate ART for all persons with HIV within 7 days of diagnosis (rapid ART) [2].

As part of the Bukoba Tanzania Combination Prevention Evaluation (BCPE), we developed a peer-delivered, linkage case management (LCM) program to provide the package of linkage services recommended by CDC and WHO. The goal of LCM was to enroll in HIV care within three months of diagnosis ≥90% of HIV-infected, out-of-care persons diagnosed in facility and community settings. At the time our intervention was developed in 2014, many reports from sub-Saharan Africa including Tanzania suggested that fewer than 50% of persons enroll early in HIV care following diagnosis, particularly persons diagnosed in community settings [7,8,11,12].

We report on the uptake, per-client program cost, and impact of LCM on early (≤90 days) enrollment in care and ART-initiation among persons diagnosed in facility and community settings during the intervention phase of BCPE. LCM services and outcomes are reported by client demographic characteristics and participation during three different ART-eligibility periods.

## Materials and methods

### Study population

BCPE is an evaluation of the population-level impact of a community-wide, integrated scale up of HIV testing and counseling (HTC), LCM, and defaulter-tracing programs in Bukoba Municipal Council, a Lake Victoria community in northwest Tanzania of approximately 150,000 persons [13]. The LCM study population included consenting eligible clients who tested HIV-positive from October 2014 through March 2017 at outpatient departments and other clinics at 11 health facilities (facility clients), and at homes, venues, or community events in all municipal wards (community clients). Excluding military and police facilities, all municipal government health facilities participated in BCPE including one regional referral hospital, two health centers, and five dispensaries. Three private, ministry-supported health centers also participated in BCPE. Clients who tested HIV-positive in accordance with the national rapid-testing algorithm were routinely screened for eligibility. Clients were eligible for LCM if they were newly or previously diagnosed and had not received HIV care in the past 90 days (HIV-positive, out-of-care). HIV-positive, out-of-care clients were ineligible for LCM if they requested referral to non-participating military or police facilities within and all facilities outside of Bukoba Municipal.

### Linkage services

#### Peer-delivered, linkage case management

LCM was delivered by peer HIV-positive, ART-adherent expert-client counselors (ECs) who received two weeks of training on HIV/AIDS and providing psychosocial support and ART adherence counseling [1,10]. ECs used standard program forms to provide and record linkage services for up to 90 days. Linkage cases could be closed before 90 days if ART-eligible clients enrolled in HIV care and returned to care at least once after ART initiation. Nurse linkage and retention coordinators (LRCs) supervised ECs ensuring that high-quality LCM services were routinely delivered, approving cases for closure, and coordinating clinical services. Cases of all clients who consented for LCM services in the last month of eligibility (March 2017) were closed by 31 May 2017.

LCM included the package of linkage services recommended by CDC and WHO including the use of trained HIV-positive paraprofessionals to deliver services; escort and one-time transportation (if needed) to HIV care and treatment clinics; treatment navigation; telephone appointment reminders, psychosocial assessment and support; and informational and motivational counseling on early enrollment in care and ART initiation, disclosure to and testing of partners and family members, and resolving real and perceived barriers to care [1,10].

#### Escort and treatment navigation

At facilities, ECs routinely escorted their clients on the day of diagnosis to co-located care and treatment clinics [1,10]. One-time transport by car with ECs was routinely offered to all community clients and those facility clients who wished to enroll in a different participating CTC [1,10]. During their client’s first CTC visit, ECs provided treatment-navigation services including introducing facility staff, providing psychosocial counseling and support, and helping clients understand the locations, content, and sequence of clinical, laboratory, and pharmaceutical services.

#### Face-to-face counseling and telephone support

Counseling was provided at the point of diagnosis, and in two subsequent face-to-face sessions typically conducted when clients returned to the CTC for HIV care. The first counseling session focused on the importance of early enrollment in HIV care and ART, and the two follow-up sessions focused separately on the importance of HIV-status disclosure and partner/family member testing, and identifying and resolving real and perceived barriers to care, respectively [1,10]. ECs called clients with cellphones to remind them of their CTC appointment, and to provide ongoing psychosocial and informational support [1,10].

#### CD4 cell-count testing and ART-initiation practices

Beginning in April 2015, point-of-care Pima CD4+ T-cell/μL enumeration (CD4 count) with same-day results was routinely provided to facility clients at the regional referral hospital and two government health centers, and later in 2015 and 2016, to clients at the three largest-volume health dispensaries. Tanzania national guidelines expanded the ART-initiation threshold twice during BCPE, resulting in the three ART-eligibility periods (CD4 count): October 2014 –December 2015 (<350); January 2016 –September 2016 (≤500); and October 2016 –May 2017 (any count, Test & Start). For clients who were ready and did not present contraindications, a 14-day course of antiretroviral drugs was routinely provided by licensed providers at the first CTC visit beginning in December 2015, and at the point of diagnosis in community settings beginning in November 2016.

#### Retraining and changes in EC compensation and LCM practices

During the CD4≤500 period, ECs were retrained on LCM service expectations, and LRCs were retrained to provide closer supervision and to help ECs manage difficult cases (e.g., calling and counseling clients who did not enroll early in HIV care). During CD4≤500 and Test & Start periods, ECs were provided a monthly monetary incentive of 10,000 Tanzania Shillings (approximately $5.00 USD) when telephone support and other monthly service indicators were met. The base monthly compensation for part-time ECs, 125,000 Tanzania Shillings (approximately $60 USD), did not change during the intervention. During Test & Start, ART-initiated community clients were routinely transported to the CTC to register for HIV care on the same day (if possible) to provide blood specimens for baseline tests, and transition case-management services from community- to facility-based ECs.

### Data collection and management

LRCs recorded delivered linkage services from program forms and clinical outcomes from CTC charts onto linkage registers maintained at each participating facility. BCPE monitoring and evaluation (M&E) staff visited each facility monthly to validate and count (compile) service and outcome indicators of cases that were closed that month. Depending on the service or clinical indicator, data were compiled by sex, age group, and HIV-diagnostic status (new or prior) and setting (facility or community). Senior investigators periodically validated monthly counts against register records, and register records against source forms including medical charts. Compiled data from monthly summary forms were analyzed using SAS 9.4 (SAS Institute Inc., Cary, NC, USA).

### Outcome measures, analyses, and costing

Enrollment in care was defined as registering for and receiving HIV medical services at least once at a CTC. LCM service and clinical outcomes were analyzed for facility and community clients separately and combined, by demographic characteristics and ART-eligibility period. Outcomes are reported as percentages for nominal and ordinal variables, and as medians and interquartile ranges (Q1-Q3) for ratio-scaled variables. Statistical tests for group differences were not used because LCM clients represent the population of interest.

Total and per-client LCM program costs were estimated from data collected retrospectively from financial records and staff interviews. Costs were estimated for personnel, travel, commodities, training, and equipment. Vehicle costs were annuitized at 3% a year over an expected useful life of five years. All costs were collected in Tanzanian Shillings (TZS), inflated to 2017 price levels using the annual Tanzania consumer price index (CPI) ratio of 1.05, 1.11 and 1.17 for 2014, 2015 and 2016, respectively, and converted to 2017 U.S. Dollars (USD) using the 2017 average market exchange rate (USD 1 = TZS 2,269.27) [14, 15]. We also estimated total and per-client costs for implementing a facility-based model that excluded transportation services and that substituted senior EC supervisors for nurse LRCs. We estimated costs for these hypothetical models because LCM (1) can be implemented in health facilities alone for clients who do not require transportation services, and (2) has been successfully implemented without employing LRC nurse supervisors [16].

### Human subjects

Provision of peer-delivered LCM services, and collection of linkage-service and clinical-outcome data were reviewed and approved by institutional review boards at the National Medical Research Institute, Dar es Salaam, Tanzania, and at Columbia University Medical Center, New York City, United States. All adult clients and parents or legal guardians of children age <15 years who participated in LCM provided written informed consent on the content and duration of LCM services, and the collection of contact, service, and clinical data. As part of their training, counselors were educated on the rights of human subjects and routinely informed clients as part of the consent process that they could refuse or discontinue LCM at any time without reducing their ability to receive the same health-care services.

## Results

Between October 2014 and March 2017, of 5213 HIV-positive, out-of-care persons identified by facility- (n = 4249) and community-based (n = 964) HTC programs, 408 (8%) requested referrals to non-participating facilities and were ineligible for LCM. Of 4805 eligible persons identified, 4273 (89%) consented to LCM. Consent rates were lower among community (82%) than facility (91%) clients, and among clients aged 15–24 years (85%) than clients of all other ages combined (90%). Consent rates were similar by sex (males, 90% vs. females, 88%), diagnostic status (new, 89%; prior, out-of-care, 90%), and ART-eligibility period (CD4<350, 88%; CD4<500, 91%; Test & Start, 89%).

Of 4273 LCM clients, complete data on linkage service and clinical outcomes were compiled on 4206 (98%). Of these clients, most were female (61%), aged 25–49 years (70%), newly HIV diagnosed (88%), and diagnosed in facilities (84%) and during the CD4<350 period (53%). Proportionally more community than facility clients were male (46% vs. 38%) and aged 15–24 years (26% vs. 19%); similar proportions of community and facility clients were newly HIV diagnosed and participated in different ART-eligibility periods (Table 1).

**Table 1:** LCM client characteristics, by setting of HIV diagnosis, Bukoba Combination Prevention Evaluation, Bukoba Tanzania, 2014–2017.^a^.

Client Characteristics	Total n (%)	Facility^b^ n (%)	Community^c^ n (%)
**Total**	4206 (100)	3538 (100)	668 (100)
**Gender**			
Male	1650 (39)	1343 (38)	307 (46)
Female	2556 (61)	2195 (62)	361 (54)
**Age in years**			
<15	148 (04)	134 (04)	14 (02)
15–24	837 (20)	664 (19)	173 (26)
25–49	2924 (70)	2484 (70)	440 (66)
>49	297 (07)	256 (07)	41 (06)
**HIV diagnostic status**^d^			
New	3717 (88)	3108 (88)	609 (91)
Prior, out of care for >90 days	487 (12)	429 (12)	58 (09)
**ART-eligibility Period**^e^			
Oct 2014-Dec 2015 (CD4<350)	2233 (53)	1876 (53)	357 (53)
Jan 2016-Sep 2016 (CD4≤500)	1221 (29)	1031 (29)	190 (28)
Oct 2016-May 2017 (Test & Start)	752 (18)	631 (18)	121 (18)

LCM, linkage case management; HIV, human immunodeficiency virus; ART, antiretroviral therapy.

^a^Client demographic data were transcribed from LCM program forms onto linkage registers maintained at 11 health facilities in Bukoba Municipality. Closed cases recorded on the registers were compiled monthly onto standard forms by the characteristics listed in the table. Percentages are of the total number of closed LCM cases, overall and by setting of diagnosis.

^b^Clients were HIV diagnosed in outpatient department and other clinics in three faith-based health centers and all eight government (excluding military/police) facilities in Bukoba Municipality, including one regional referral hospital, two health centers, and five dispensaries.

^c^Clients were HIV diagnosed through home- and venue-based HIV testing conducted in all municipal wards. Venues included high-traffic urban locations, clubs, and businesses.

^d^Based on client report at the time of LCM eligibility screening. Prior, out-of-care for >90 days includes persons who were previously HIV diagnosed but had never enrolled in HIV care.

^e^Changes in national ART recommendations based on patient CD4 count/μL; Test & Start = ART for all HIV-infected persons regardless of CD4 count. Dates reflect time periods in which clients received LCM services, enrollment in LCM ended 31 March 2017.

### Service uptake

Most clients received recommended services including counseling on the importance of early enrollment in care and ART initiation (100%), CTC escort (83%), treatment navigation (94%), telephone support (77% among clients with cellphones), and counseling on disclosure and partner/family testing (77%) and on real and perceived barriers to HIV care (69%). Receipt of LCM services was similar by client gender (Table 2). Proportionally more facility than community clients were escorted to CTCs on foot (79% vs. 3%), and proportionally fewer were escorted by car (9% vs. 54%). Of clients who received treatment navigation services, 83% were escorted to the CTC on foot or by car, and 11% (8% facility, 25% community) were met at the CTC by appointment. In all ART-eligibility periods, proportionally more facility than community clients received follow-up counseling on disclosure and partner/family testing, and on real and perceived barriers to HIV care (Table 2).

**Table 2:** Peer-delivered LCM service uptake, by client gender, setting of HIV diagnosis, and ART-eligibility period, Bukoba Combination Prevention Evaluation, Bukoba Tanzania, 2014–2017.^a^.

Characteristic^a^	HIV-Care Counseling^b^ n (%)	CTC Escort^c^ n (%)	Treatment Navigation^d^ n (%)	Telephone Support^e^ n (%)^f^ (%)^g^	Disclosure Counseling^h^ n (%)	Barriers Counseling^i^ n (%)
**Total**	4206 (100)	3481 (83)	3939 (94)	2726 (77) (65)	3256 (77)	2882 (69)
**Sex**						
Male	1650 (100)	1410 (85)	1565 (95)	1063 (76) (64)	1288 (78)	1125 (68)
Female	2556 (100)	2071 (81)	2374 (93)	1663 (77) (65)	1968 (77)	1757 (69)
**Diagnostic setting**						
Facility	3538 (100)	3095 (87)	3386 (96)	2312 (77) (65)	2842 (80)	2525 (71)
Community	668 (100)	386 (58)	553 (83)	414 (77) (62)	414 (62)	357 (53)
**ART-eligibility period**^j^						
***Facility***						
Oct 2014-Dec 2015 (CD4<350)	1876 (100)	1538 (82)	1753 (93)	1023 (64) (55)	1430 (76)	1316 (70)
Jan 2016-Sep 2016 (CD4<500)	1031 (100)	960 (93)	1016 (99)	821 (92) (80)	896 (87)	760 (74)
Oct 2016-May 2017 (Test & Start)	631 (100)	597 (95)	617 (98)	468 (89) (74)	516 (82)	449 (71)
***Community***						
Oct 2014-Dec 2015 (CD4<350)	357 (100)	176 (49)	282 (79)	199 (69) (56)	222 (62)	194 (54)
Jan 2016-Sep 2016 (CD4≤500)	190 (100)	97 (51)	155 (82)	133 (90) (70)	126 (66)	109 (57)
Oct 2016-May 2017 (Test & Start)	121 (100)	113 (93)^k^	116 (96)	82 (83) (68)	66 (55)	54 (45)

LCM, linkage case management; HIV, human immunodeficiency virus; ART, antiretroviral therapy; CTC, HIV care and treatment clinic.

^a^Trained HIV-positive expert client peer counselors provided the six types of services recommended by the Centers for Disease Control and Prevention and the World Health Organization during a maximum 90-day case management period. Client demographic and linkage-service data were transcribed from LCM program forms onto linkage registers maintained at 11 health facilities in Bukoba Municipality. Service data were compiled monthly from closed cases onto standard forms by the characteristics listed in the table. Service indicators were not compiled by age group. Percentages are of the total number of LCM clients with the characteristic of interest.

^b^On the day of participation and often in subsequent counseling sessions, all LCM clients were counseled on the importance of early enrollment in HIV care and ART initiation and adherence, if eligible.

^c^Includes either being escorted on foot or transported by car with expert client counselor from the diagnostic setting to the referral CTC. At facilities, clients were typically escorted from the outpatient department clinic to the co-located CTC. One-time transportation services were routinely offered to all community clients and clients at facilities who wished to enroll in care at a different CTC. Some community outreach events were conducted near a municipal HIV clinic, permitting escort to the CTC on foot.

^d^Accompanied by expert client counselor for the duration of the first CTC visit, and receiving psychosocial support and informational counseling on the content, sequence, and location of CTC services. If not escorted by foot or car, clients were met at the CTC by appointment.

^e^Spoke with expert client counselor at least once by phone during the linkage case management period. Telephone calls were conducted to assess wellbeing, remind clients of upcoming CTC appointments, and provide psychosocial support, and informational and motivational counseling.

^f^Of 3543 (84%) clients who provided a cellphone number including 3006 (85%) and 537 (80%) facility and community clients, respectively. During the CD4<350, CD4≤500, and Test & Start periods, 1883 (84%), 1037 (85%), and 623 (83%) clients provided a cellphone number, respectively.

^g^Of all clients.

^h^Provided typically during a return visit to the CTC. A standard form was used to assess client HIV-status disclosure, and HIV test outcomes of partners and family members of clients.

^i^Provided typically within 2 weeks after the first follow-up session during a return visit to the CTC. A standard form was used to assess 12 types of barriers to care so that counseling could be tailored to meet individual needs.

^j^Changes in national ART recommendations based on patient CD4 count/μL; Test & Start = ART for all HIV-infected persons regardless of CD4 count. Dates reflect time periods in which clients received LCM services, enrollment in LCM ended 31 March 2017.

^k^During Test & Start, point-of-diagnosis, same-day ART initiation was routinely provided during community testing events beginning 8 November 2016. All community clients initiated on ART were routinely provided immediate, one-time transportation to CTCs to complete baseline clinical assessment and laboratory tests, and ensure to the extent possible continuum of facility-based care. If CTCs were not open at the time of community testing, all clients were encouraged to make an appointment for one-time transportation services on another day.

Compared with the CD4<350 period, proportionally more clients in CD4≤500 and Test & Start periods received CTC escort, treatment navigation, and telephone-support services (Table 2). During Test & Start, nearly all facility and community clients received CTC escort (95% and 93%, respectively) and treatment navigation (98% and 96%, respectively) services. Of facility and community clients with cellphones (85% and 80%, respectively), a large majority were contacted by phone at least once in the CD4≤500 and Test & Start periods, and the monthly median (IQR) telephone contacts per client increased from 1.3 (0.8–2.0) during the CD4<350 period to 3.7 (2.8–4.5) during Test & Start.

#### Partner and family member testing and barriers to care

Of 3256 clients who completed the session on disclosure, 1617 (50%) reported having a main partner, and of 229 partners tested for HIV as part of LCM services, 120 (52%) were newly HIV diagnosed. An additional 462 family members of clients also tested for HIV as part of LCM services, of whom 56 (12%) were newly HIV diagnosed. Of 2882 facility and community clients who completed the session on barriers to HIV care, proportionally more community than facility clients (43% vs. 37%) reported at least one barrier to care. Similar proportions of female (39%) and male (36%) clients reported at least one barrier to care.

### Enrollment in HIV care

Within 90 days of HIV diagnosis, 93% of all clients, and 95% of 3538 facility and 82% of 668 community clients enrolled in HIV care. From CD4<350 to Test & Start periods, enrollment in care increased from 93% to 98% and from 78% to 96% among facility and community clients, respectively (Table 3). Of 752 clients during Test & Start, 98% and 97% of males (n = 310) and females (n = 442), and 96% and 98% of clients aged 15–24 (n = 150) and 24–49 (n = 535) years, respectively, enrolled in care.

**Table 3:** Enrollment in HIV care within 90 days of LCM program consent, by setting of HIV diagnosis and client characteristics, Bukoba Combination Prevention Evaluation, Bukoba Tanzania, 2014–2017.^a^.

	All Clients		Facility		Community	
Client Characteristics	Total n	Enrolled^b^ n (%)	Total n	Enrolled^b^ n (%)	Total n	Enrolled^b^ n (%)
**Total**	4206	3918 (93)	3538	3367 (95)	668	551 (82)
**Gender**						
Male	1650	1561 (95)	1343	1298 (97)	307	263 (86)
Female	2556	2357 (92)	2195	2069 (94)	361	288 (80)
**Age in years**						
< 15	148	145 (98)	134	132 (99)	14	13 (93)
15–24	837	737 (88)	664	606 (91)	173	131 (76)
25–49	2924	2751 (94)	2484	2377 (96)	440	374 (85)
> 49	297	285 (96)	256	252 (98)	41	33 (80)
**HIV diagnostic status**^c^						
New	3717	3462 (93)	3108	2960 (95)	609	502 (82)
Prior, out-of-care >90 days	487	456 (94)	429	410 (96)	58	46 (79)
**ART-eligibility period**^d^						
Oct 2014-Dec 2015 (CD4<350)	2233	2018 (90)	1876	1738 (93)	357	280 (78)
Jan 2016-Sep 2016 (CD4≤500)	1221	1168 (96)	1031	1013 (98)	190	155 (82)
Oct 2016-May 2017 (Test & Start)	752	732 (97)	631	616 (98)	121	116 (96)

LCM, linkage case management; HIV, human immunodeficiency virus; ART, antiretroviral therapy; CTC, HIV care and treatment clinic.

^a^Client demographic and HIV clinical data were transcribed from LCM program forms and CTC medical records onto linkage registers maintained at 11 health facilities in Bukoba Municipality. Closed cases recorded on the registers that enrolled in HIV care were compiled monthly onto standard forms by the characteristics listed in the table. Percentages are of the total number of LCM clients with the characteristic of interest, overall and by setting of HIV diagnosis.

^b^Client received HIV care at a fixed standing CTC at least once, confirmed by medical chart review.

^c^Based on client report at the time of LCM eligibility screening. Prior, out-of-care for >90 days includes persons who were previously HIV diagnosed but never enrolled in HIV care.

^d^Changes in national ART recommendations based on patient CD4 count/μL; Test & Start = ART for all HIV-infected persons regardless of CD4 count. Dates reflect time periods in which clients received LCM services, enrollment in LCM ended 31 March 2017.

#### ART eligibility and initiation among clients enrolled in HIV care

Before Test & Start, of 3186 clients who enrolled in HIV care, 3032 (95%) overall, and 95% and 94% of facility and community clients, respectively, had a baseline CD4 test result recorded in the linkage register. During CD4<350 and CD4≤500 periods, of clients who enrolled in HIV care on whom CD4 test results were available, 48% and 71%, respectively, were ART eligible by CD4 count alone. During CD4<350, CD4≤500, and Test & Start periods, of all 3918 clients who enrolled in care, 52%, 70%, and 89%, respectively, were initiated on ART within 90 days of diagnosis. Proportionally more enrolled facility than community clients were ART eligible by CD4 count and initiated on ART overall and during both CD4<350 and CD4≤500 periods (Table 4).

**Table 4:** ART initiation within 90 days of LCM program consent, by setting of HIV diagnosis and client characteristics, Bukoba Combination Prevention Evaluation, Bukoba Tanzania, 2014–2017.^a^.

	All Clients		Facility		Community	
Client Characteristic	ART Eligible^b^ (%)	ART Initiated^c^ n (%)^d^ (%)^e^	ART Eligible^b^ (%)	ART Initiated^c^ n (%)^d^ (%)^e^	ART Eligible^b^ (%)	ART Initiated^c^ n (%)^d^ (%)^e^
**Total**	(64)	2521 (64) (60)	(66)	2247 (67) (64)	(51)	274 (50) (41)
**Gender**						
Male	(71)	1025 (66) (62)	(74)	878 (68) (65)	(58)	147 (56) (48)
Female	(59)	1496 (63) (59)	(61)	1369 (66) (62)	(44)	127 (44) (35)
**Age in years**						
< 15	(100)^f^	122 (84) (82)	(100)^f^	111 (84) (83)	(100)^f^	11 (85) (79)
15–24	(51)	399 (54) (48)	(51)	350 (58) (53)	(47)	49 (37) (28)
25–49	(68)	1799 (65) (62)	(70)	1613 (68) (65)	(51)	186 (50) (42)
> 49	(72)	201 (71) (68)	(72)	173 (69) (68)	(79)	28 (85) (68)
**ART-eligibility period**^g^						
Oct 2014-Dec 2015 (CD4<350)	(48)	1057 (52) (47)	(50)	971 (56) (52)	(32)	86 (31) (24)
Jan 2016-Sep 2016 (CD4≤500)	(71)	815 (70) (67)	(73)	732 (72) (71)	(60)	83 (54) (44)
Oct 2016-May 2017 (Test & Start)	(100)	649 (89) (86)	(100)	544 (88) (86)	(100)	105 (91) (87)

LCM, linkage case management; HIV, human immunodeficiency virus; ART, antiretroviral therapy; CTC, HIV care and treatment clinic.

^a^Client demographic and HIV clinical data were transcribed from LCM program forms and CTC medical records onto linkage registers maintained at 11 health facilities in Bukoba Municipality. Closed cases recorded on the registers that were initiated on ART were compiled monthly onto standard forms by the characteristics listed in the table. ART initiation was not compiled by HIV diagnostic status.

^b^By CD4 count alone, based on national ART recommendations in effect at the time of case closure. Before Test & Start, percentages are of all clients who enrolled in HIV care on whom a CD4 count was recorded in the linkage register (Table 3). Of 3186 clients who enrolled in HIV care before Test & Start, 3032 (95%) clients overall, and 2622 (95%) and 410 (94%) facility and community clients, respectively, had a baseline CD4 test result in the linkage register. During Test & Start, all clients were ART eligible by CD4 count alone.

^c^Recorded from CTC medical charts.

^d^Of LCM clients enrolled in HIV care (Table 3).

^e^Of all LCM clients (Table 3).

^f^Based on Tanzania National Guidelines for the Management of HIV and AIDS, May 2015. Before May 2015, children <15 years of age were eligible for ART if they met specific CD4 percentage or CD4 count/μL criteria.

^g^Changes in national ART recommendations based on patient CD4 count/μL; Test & Start = ART for all HIV-infected persons regardless of CD4 count. Dates reflect time periods in which clients received LCM services, enrollment in LCM ended 31 March 2017.

#### ART initiation among all clients and during test and start

Of all 4206 clients, 2521 (60%) initiated ART within 90 days of diagnosis, including: 47%, 67%, and 86% of clients during the CD4<350, CD4≤500, and Test & Start periods, respectively (Table 4). Of 752 clients during Test & Start, 86% and 87% of facility and community clients, 85% and 87% of males and females, and 79% and 87% of clients aged 15–24 and 25–49 years, respectively, initiated ART. Among 752 clients who participated in LCM during the first (October–December, 2016, n = 289) and second (January–March, 2017, n = 463) quarters of the rollout of Test & Start, 79% and 91% initiated ART, respectively.

### LCM program costs

The estimated total LCM program cost was $186,860 USD, with most costs incurred for personnel (62%), followed by travel and transport services (24%), commodities and supplies (6%), training (6%), and equipment (1%) (Table 5). The estimated per-client program cost ($44 USD) was relatively stable across ART-eligibility periods: CD4<350, $45 USD; CD4≤500, $46 USD; and Test & Start, $40 USD. Most personnel costs were attributed to nurse LRCs (70%). An LCM model substituting senior ECs for nurse LRCs would reduce personnel costs to $48,744 USD, total costs to $119,079 USD, and per-client costs to $28 USD. Implementing this EC-managed LCM model at health facilities without providing transportation services (excluding driver, vehicle, and fuel costs) would further reduce total costs to $74,591 and per-client costs to $18 USD.

**Table 5:** Total and average monthly LCM program costs (USD), by category and ART eligibility period, Bukoba Combination Prevention Evaluation, Bukoba Tanzania, 2014–2017.^a^.

	All Periods			ART Eligibility Period					
	Oct 2014 –May 2017			Oct 2014–Dec 2015		Jan 2016–Sep 2016		Oct 2016–May 2017	
	(CD4<350 –Test & Start)			(CD4<350)		(CD4≤500)		(Test & Start)	
Category	Total USD^b^	USD/Month^b^^,^^c^	%^b^	USD/Month^c^	%^b^	USD/Month^b^^,^^c^	%	USD/Month^c^	%^b^
**Total**	186,860	6,229	100	6,694	100	6,235	100	5,055	100
Personnel^d^	116,525	3,884	62	4,156	62	3,806	61	3,324	66
Travel^e^	44,489	1,483	24	1,475	22	1,488	24	1,494	30
Commodities^f^	11,909	397	6	368	5	552	9	237	5
Training	11,681	389	6	695	10	139	2	-	
Equipment^g^	2,257	75	1	0	0	251	4	-	

LCM, linkage case management; ART, antiretroviral therapy; USD, United States dollar.

^a^All costs were collected in Tanzanian Shillings (TZS) and inflated to 2017 price levels using the Tanzania consumer price index at an average rate of 1.05, 1.11, and 1.17 per year for 2014, 2015, and 2016 respectively. After inflating to 2017 price levels, costs were converted to USD using the average market exchange rate in 2017 (USD 1 = TZS 2269.27).

^b^Categories do not sum to total or 100% due to rounding error.

^c^Average monthly cost = total cost / months of participant recruitment. Total participant recruitment period = 30 months; CD4<350 period = 15 months; CD4<500 period = 9 months; Test & Start period = 6 months. LCM enrollment ended 31 March 2017 and LCM services ended 31 May 2017.

^d^Expert client counselors, nurse linkage and retention coordinators, and nurse counselors.

^e^Includes costs for drivers, vehicles, and fuel. Costs for vehicles were annuitized at 3% a year over an expected useful life of five years.

^f^Includes office stationary and wireless access points.

^g^Includes laptops and office furniture.

## Discussion

Of over four thousand persons HIV diagnosed in facility and community settings in Bukoba Municipality who participated in a peer-delivered, linkage-case-management program, nearly all received linkage services recommended by CDC and WHO, and within three months of diagnosis, most (93%) enrolled in HIV care and many (60%) were initiated on ART during a period (October 2014 –May 2017) when ART-eligibility thresholds were expanded from CD4<350 to Test & Start. Although proportionally fewer community than facility clients received linkage services and enrolled early in HIV care, delivery and uptake of escort, treatment navigation, and telephone-support services improved over time. During Test & Start, early enrollment in HIV care and ART initiation exceeded 95% and 85%, respectively, for both facility and community clients.

Our finding of near universal early enrollment in care among LCM clients are similar to those reported from a peer-delivered LCM program in Eswatini that also provided the package of CDC/WHO recommended services [16], and stands in contrast to findings from studies conducted in sub-Saharan Africa on persons who are provided a referral as the only linkage service following HIV diagnosis. In fifteen studies conducted before Test & Start, for example, only 18–51% and 39–71% of persons diagnosed in community and facility settings, respectively, enrolled early in HIV care following referral [7]. Even with expansion of ART eligibility during Test & Start, when referral to care is the only linkage service, reported early enrollment often falls well below 90%, particularly among community clients [17–20]. Notably, in two large combination prevention trials in South Africa and Zambia, of the nearly 7,000 intervention-arm participants who were informed that they would be initiated on ART, only 37% and 41%, respectively, enrolled in care within three months of diagnosis following referral alone, and <50% in both studies had initiated ART by six months of diagnosis [19,20].

Consistent with a systematic review and more recent studies in Tanzania, proportionally fewer community than facility clients before Test & Start were ART-eligible by CD4 count, and proportionally fewer were initiated on ART [7,17,18]. Because community clients at the time of participation were not seeking health care and were less likely to be ART eligible, they may have been less likely than facility clients to perceive the need to enroll early in HIV care [17,18,21–23]. Including point-of-care CD4 testing as part of linkage services might have helped some community clients overcome this important barrier to care before Test & Start [24–25].

Not surprisingly, when point-of-diagnosis ART-initiation combined with same-day transport and treatment navigation services were routinely provided during Test & Start, similarly high proportions of community and facility clients initiated ART and enrolled in HIV care. Encouragingly, in the last three months of the rollout of Test & Start, >90% of over 450 facility and community clients were initiated on ART. Our findings suggest that escort, treatment navigation, and point-of-diagnosis ART initiation can achieve near universal rapid ART initiation and enrollment in facility-based care among persons diagnosed in community settings. Rapid ART initiation within seven days diagnosis is now recommended for all persons without contraindications who are ready to begin ART [2,26].

As recommended by CDC and WHO, peer-delivered services were initiated for all consenting clients at the point of diagnosis [1,10]. Linkage programs that require either referral forms or documented missed appointments to initiate follow-up may lose important opportunities to provide timely and effective linkage services [8,20]. In KwaZulu-Natal, for example, of over 1,000 persons diagnosed at home who were supposed to receive linkage services after failing to present for care within three months, enrollment in care 3 and 6 months after diagnosis increased from only 37% to 47% [20]. As a proactive program, LCM expert client counselors initiate services at diagnosis to build rapport, assess and understand individual circumstances, and use their personal experiences in living with HIV to help clients cope with their disease and overcome barriers to HIV care [27–29]. These proactive, peer-delivered services may be particularly helpful to younger persons and men who are more likely to delay their enrollment in care because of real or perceived barriers to HIV care [1–3,7–9].

The importance of routinely assessing and mitigating barriers to care is underscored by the considerable magnitude and multidimensionality of reported barriers, and in a recent study, that persons who report one or more barriers have significantly increased mortality one year after diagnosis [1,10,21–23,27–33]. During Test & Start, nearly a third of enrolled clients who completed the third counseling session reported at least one barrier to care.

ECs were expected to identify and help resolve barriers to care during all counseling sessions. However, because we routinely assessed barriers in the third session, usually conducted several weeks after enrollment in care, many clients did not receive the assessment and our findings on the proportion of clients with at least one barrier is likely an underestimate. Reasons for the decrease in the delivery or uptake of the barriers counseling session are unclear, but might be attributed, in part, to clients who did not keep their CTC appointment. To identify and mitigate barriers throughout the entire LCM period, barriers should be routinely identified and addressed during all linkage sessions as part of an individual-level M&E system. While our standard form assessed 12 specific barriers to help target counseling, we could not monitor and evaluate the frequency and distribution of specific barriers with our register-based system.

Non-disclosure of HIV status is a particularly important barrier to both enrollment and retention in HIV care [17,18,21,23,27–32]. Integrated as part of LCM, counseling on the importance of HIV-status disclosure and partner and family-member testing was provided to most clients. Although our evaluation was unable to assess the efficacy of counseling on disclosure practices, ECs reported helping many clients disclose to and test partners and family members. Consistent with many studies, of the nearly 700 partners and family members who tested through LCM services, many were newly HIV diagnosed, including approximately half of over 200 partners tested [34–37]. Our finding on high yield of new diagnoses but relatively low uptake by partners underscores the importance of fully implementing LCM partner-notification services. Greater uptake of these services might be achieved by adopting best practices from other studies and ensuring that all recommended partner elicitation and notification strategies are routinely employed [34–38].

Studies conducted in sub-Saharan Africa that have reported costs for linkage services including point of diagnosis CD4 testing, transportation assistance, and follow-up telephone or face-to-face counseling also included HTC-related costs for identifying HIV-positive clients (per-client costs in these studies ranged from $111 to $2102 USD) [39–42]. Because HTC-related costs were included in these studies, comparative linkage-program costs are not available. Based on our experiences in Bukoba and elsewhere, reducing LCM service costs while maintaining >90% early enrollment in care is possible. A similar LCM program in Eswatini did not employ nurses to supervise ECs and enrolled in HIV care 98% of 651 clients [16]. In health facilities, implementing an EC-managed model without travel and transportation-service costs would enable routine delivery of peer-delivered, LCM services at relatively low cost (~$18 USD per client with similar personnel-compensation, training, commodity, and equipment costs). Not providing transportation services for facility clients should have minimal impact on enrollment in care as few clients requested transportation services to another ART clinic.

Although a facility-based, escort-only model might achieve similarly high enrollment in care rates at even lower per-client costs, peer-delivered LCM might have additional durable effects such as reducing barriers associated with defaulting from ART care [27–29]. Given CDC and WHO recommendations to routinely address barriers to care, particularly non-disclosure, further research is needed to identify optimal methods for integrating barrier assessment and mitigation as part of LCM services, and evaluating corresponding costs and effects on both enrollment and retention in ART care [1,10].

Findings in this report are subject to several additional limitations not previously mentioned. First, some linkage-service and clinical data may not have been recorded completely or accurately onto linkage registers. Senior-investigator audits throughout the intervention period, however, found that register data consistently matched source forms, including patient medical records. Second, although manual compilation of outcomes is bound to include some errors, quality-assurance procedures routinely prompted re-compilations that identified few and relatively minor discrepancies. Nonetheless, outcomes of approximately 2% of consented clients either were not compiled or compilation of consented records were in error. Third, although consent rates were high, not all eligible persons chose to participate in LCM, particularly clients who were younger and diagnosed in the community. Enrollment in HIV care among LCM non-participants is unknown. Fourth, ART initiation was measured during the 90-day LCM period only. After Test & Start was approved, however, a comprehensive call-back and defaulter-tracing program was conducted as a good-faith effort to inform and initiate on ART all clients who were not initiated on ART in the 90-day LCM period. Finally, because our community-wide program evaluation did not include a control group, we could not estimate LCM intervention effects and improvements in services on enrollment in care overall and by time period. Although our enrollment in care findings far exceed most historical cohorts in Tanzania and elsewhere in sub-Saharan Africa, observed differences may also be attributed to increased awareness of the benefits and availability of ART and improvement in HIV-related societal norms. LCM enrollment-in-care outcomes, however, were substantially higher than historical cohorts in the CD4<350 era in which most cohorts were evaluated, and remain higher compared with several cohorts in the Test & Start era [7,8,11,12,17–20].

We found that implementing the package of CDC and WHO recommended linkage services was feasible and well accepted, and during Test & Start, achieved near universal early enrollment in HIV care and ART initiation at modest cost. In 2017, peer-delivered LCM services was approved by the Tanzania Ministry of Health, Community Development, Gender, Elderly, and Children as a new service delivery model, and in 2018, was implemented as facility- and community-based linkage interventions in ten regions in Tanzania by four HIV prevention organizations [43]. As a recommended solution by the United States President’s Emergency Plan for AIDS Relief, peer-delivered LCM services should be considered for implementation in other countries and programs with <90% early enrollment into ART care among persons diagnosed in facility and community settings [44].

## Supporting information

S1 Tables LCM consent, linkage service, and clinical outcomes. (XLSX)

S2 Tables LCM costing tables. (XLSX)
