# Correction: Implementing the package of CDC and WHO recommended linkage services: Methods, outcomes, and costs of the Bukoba Tanzania Combination Prevention Evaluation peer-delivered, linkage case management program, 2014-2017

**DOI:** 10.1371/journal.pone.0210048

**Published:** 2018-12-27

**Authors:** The PLOS ONE Staff

## Notice of republication

This article was republished on December 17, 2018 to correct an error in the title introduced during the typesetting process. The publisher apologizes for the error. Please download this article again to view the correct version. The originally published, uncorrected article and the republished, corrected article are provided here for reference.

## Supporting information

S1 File Originally published, uncorrected article. (PDF)

S2 File Republished, corrected article. (PDF)
